# Knowledge, perception and attitude of patient safety amongst clinical year physiotherapy students in Ghana

**DOI:** 10.4102/sajp.v77i1.1499

**Published:** 2021-02-19

**Authors:** Samuel-Jerry S. Atakora, Jonathan Quartey, Samuel K. Kwakye

**Affiliations:** 1 Department of Physiotherapy, Komfo Anokye Teaching Hospital, Kumasi, Ghana; 2 Department of Physiotherapy, School of Biomedical and Allied Health Sciences, College of Health Sciences, University of Ghana, Accra, Ghana; 3 Department of Physiotherapy, West Africa Football Academy, Sogakope, Ghana

**Keywords:** physiotherapy, education, clinical year, healthcare, human error, patient safety

## Abstract

**Background:**

Patient safety is a part of healthcare that is not only important in the delivery of healthcare but also in the training of healthcare professionals. It is a key component of physiotherapy treatment which, when underrated, can result in more harm than good.

**Objective:**

To determine the level of knowledge, perception and attitude of patient safety amongst physiotherapy students.

**Method:**

Eighty clinical year physiotherapy students from the University of Ghana and the University of Health and Allied Sciences were recruited for this cross-sectional study. Data were obtained using the World Health Organization Medical School Curricular Guide for Patient Safety questionnaire. Pearson Chi-square was used to test for association between the level of study of participants and their knowledge on patient safety.

**Results:**

Of the eighty (80) respondents, there were 41 women (52.1%) and 39 men (48.8%) in our study. Majority of the respondents (97.5%) had a moderate level of knowledge on patient safety. There was no significant association between the level of study and knowledge of clinical year physiotherapy students on patient safety (*p* = 0.712).

**Conclusion:**

Clinical year physiotherapy students in Ghana have a moderate level of knowledge on the concept of patient safety. Restructuring of the physiotherapy curriculum to specifically cover the concept of patient safety would be beneficial to its promotion in the healthcare system.

**Clinical implications:**

The outcomes of our study may motivate physiotherapy students to put in additional effort that could facilitate the translation of positive attitudes that have been shown to be effective in reducing errors and promoting patient safety.

## Introduction

The World Health Organization defines patient safety ‘as the absence of preventable harm to a patient during the process of healthcare and reduction of risk of unnecessary harm associated with healthcare to an acceptable minimum’ (World Health Organization 2018).

In recent times, the main foci of healthcare leaders and stakeholders are the quality and safety of healthcare provided (Cresswell et al. 2013). The complex nature of patient safety has made its maintenance challenging for healthcare professionals and hospital managers. Estimates of current prevalence vary, but it is widely considered that up to 10% of hospitalised patients suffer some form of unintentional harm or an adverse event; with most deemed preventable (AIHW 2016; NHS Scotland 2016).

One factor that contributes to health professionals’ competence is safe practice. Healthcare-associated harm is mostly because of actions taken and/or plans made during rendering healthcare services, rather than what is associated with an underlying disease or injury (Yardley et al. 2018). Rodziewicz, Houseman and Hipskind (2020) argue that unexpected harm to a patient may arise from professional, organisational and system factors. Usher et al. (2017) suggested that professional factors such as knowledge, attitude and skills influence patient safety. Issues of forgetfulness, inattention, carelessness, poor motivation, sense of fear, writing procedures, malpractice, the threat of litigation and disciplinary measures are all unsafe acts which expose patients to preventable harms (Castel et al. 2015). The exposure of patients to preventable harm may be because of ‘inadequate training, lack of clinical skills, lack of supervision of clinical staff, and the failure to follow policies or protocols’ (Ker 2011).

To achieve the promotion and improvement of patient safety, there must be a strong and positive patient safety culture in the healthcare setting, and this ought to be given top priority (Bagnasco et al. 2011; Dekker & Breakey 2016). All groups of healthcare professionals, including physiotherapists play a critical role in ensuring and maintaining patient safety. Physiotherapists’ responsibilities do not only deal with direct patient interaction but also the health settings and systems within which care is provided (Canadian Physiotherapy Association 2019).

Struessel, Rodriguez and Van Zytveld (2017) state that irrespective of the highest quality of training received by a healthcare professional anywhere in the world, he or she can never become exempted from error. Invariably, it means that during the practice of physiotherapy in Ghana, mistakes and errors will be inevitable. Rosenfeld (2017) lists some common practices that compromise the safety of patients during physiotherapy management as follows: failing to properly teach patients how to use equipment, leaving patients unsupervised during exercises, failure to listen to patients’ complaints of pain, failing to repair equipment in a physiotherapy centre and allowing patients to fall during exercise. Rosenfeld (2017) further suggests that concussion, broken bones, anxiety, heart attack and need for additional revision of surgeries could be the consequences when patient safety is neglected.

The concept of patient safety motivates healthcare professionals, including physiotherapists, to choose behaviours that improve and not reduce the safety of patients (Fleming 2005). Providing healthcare professionals with knowledge, skills and positive attitudes towards patient safety, is expected to enhance safe practices, improve patient care and reduce the rate of morbidity and mortality (Schnall et al. 2008).

Brasaite, Kaunonen and Suominen (2015) report that having a suitable knowledge base is central to the quality of any profession and therefore the more awareness and knowledge of patient safety present in healthcare organisations, the more it will be practiced. For this reason, many healthcare educational programmes have clinical placements where students visit health centres to familiarise themselves with the hospital environment as well as clinical procedures (Nsiah-Asare 2017). Medical students’ knowledge of patient safety is low but formal or informal education on patient safety topics is associated with their increased knowledge (Blasiak et al. 2014). Usher et al. (2017) report a high knowledge of patient safety amongst nursing students. According to Usher et al. (2017), nursing students agree that the broader aspects of patient safety in health professions education are well covered in their education, which further supports the findings of Blasiak et al. (2014) concerning medical students.

Students attain detailed experience during clinical placements, which are most often supervised. These placements provide students with the opportunity to observe, reflect, form abstract concepts, and practice new skills (Kolb & Kolb 2005). Doyle et al. (2015) and Stevanin et al. (2015) have explored the knowledge of patient safety and skills of undergraduate nursing, medical and pharmacy students, and the practice of beginning level health professionals. However, it appears that few studies that specifically concern patient safety knowledge amongst undergraduate physiotherapy students have been conducted. Physiotherapy students whilst on clinical rotations amongst other things learn the concept of patient safety to enable them to reduce the risk of error by the time they begin to practice. For this reason, the need to determine the knowledge of patient safety prior to graduation in the 4-year BSc Physiotherapy programmes at two universities in Ghana is important.

The objective of our study was therefore to determine the level of knowledge, perception and attitude of patient safety amongst physiotherapy students at the above two universities.

## Method

Our cross-sectional study involved third and fourth year (levels 300 and 400) clinical year physiotherapy students of the University of Ghana (UG) and the University of Health and Allied Sciences (UHAS). Undergraduate physiotherapy education in Ghana is a Bachelor of Science degree earned after 4 years of training. The first 2 years is the preclinical phase of training, which involves mostly classroom theory, whilst the clinical phase, which is also another 2 years is a combination of classroom theory and clinical practice (theory and practice). The clinical practice is conducted under the supervision of experienced clinicians and academic staff at certified physiotherapy facilities that are mostly state owned.

A sample size of 79 was calculated using the Taro Yamane’s formula, *n* = N/1 + N (e^2^) (Yamane 1967) from a total population of 98 levels 300 and 400 students from both universities of which 52 are from UG and 46 from UHAS. First and second year (levels 100 and 200) preclinical physiotherapy students were excluded from our study.

### Instrument for data collection

The World Health Organization Medical School Curricular Guide for Patient Safety questionnaire (Appendix I) was adapted by replacing ‘doctors’ with ‘physiotherapists’ and ‘nurses’ with ‘physiotherapy assistants’ and was utilised to collect data (World Health Organization 2009). It is divided into five sections, namely ‘error and patient safety’ which consists of seven items; ‘safety of the healthcare system’ which has six items; ‘personal influence over safety’ which has seven items as well as ‘personal attitudes to patient safety’ which consists of four items. The fifth section has nine items which deal with ‘safety at the workplace’. The questionnaire is scored on a five-point Likert scale which commences from one indicating ‘strongly disagree’, to 5 for ‘strongly agree’ for the items. Scores were classified as low, moderate and high levels with designated corresponding ranges (Appendix II). A test-retest amongst 20 clinical year physiotherapy students who did not take part in our study, for reliability and feasibility purposes was conducted and yielded a Cronbach’s alpha (α) of 0.74 was realised. A data capturing form was designed to obtain demographic data of participants.

### Procedure for data collection

Introductory letters to the heads of department of the two universities were obtained from the School of Biomedical and Allied Health Sciences (UG) and delivered. The rationale and nature of the study were fully explained to the participants and informed that participation was voluntary. Participants were enrolled using convenience sampling. Copies of the questionnaire, which had only identifying code numbers of the enrolled participants to ensure anonymity and takes approximately 20 min to complete, were distributed to participants in their respective schools by the authors. Completed copies of the questionnaire were retrieved on the same day whilst the authors did daily follow ups for 2 weeks to retrieve the remaining questionnaire from respondents at the University of Ghana. Respondents from UHAS were also visited on two weekends to retrieve available completed copies of the questionnaire. The instrument is designed to be administered before and after the different aspects of patient safety which is covered in training, however this aspect appears not to be included in the undergraduate physiotherapy curriculum, hence it was administered once based on the students’ practical knowledge acquired during training.

### Data analysis

Collected data were entered into Microsoft Excel version 2016 and analysed using SPSS version 23. A Pearson Chi-square test was used to test for associations between the level of study of participants and their knowledge on patient safety at a significance level of 0.05.

### Ethical consideration

Ethical clearance (SBAHS – PT./10563067/SA/2018-2019) was obtained from the Ethics and Protocol Review Committee of the School of Biomedical and Allied Health Sciences. Permission was sought from the heads of the physiotherapy departments of the two universities. Participants signed informed consent forms before participating in our study.

## Results

A total of 80 clinical year physiotherapy students was recruited. Thirty-nine (48.8%) of the respondents were men and 50 (62.5%) were level 300 students. Twenty-seven (33.75%) respondents were level 300 UHAS students, whilst 14 (17.5%) were level 400 UG students.

The majority (70%) of the respondents showed a moderate level (15–27) of knowledge about ‘error and patient safety’, 10 (12.5%) indicated a low (7–14), and the remaining 14 (17.5%) showed high (28–35) levels. Forty-eight (60%) of the respondents indicated a high level (19–30) of knowledge on safety of the healthcare system, whilst 32 (40%) showed a moderate level (13–18). Table 1 depicts the responses to knowledge on safety of the healthcare system. With regards to knowledge on personal influence and attitudes to patient safety, 57 (71.25%) respondents showed moderate (23–33) and 23 (28.75%) showed high (34–55) levels. Table 2 depicts the outcomes on personal influence and attitudes to patient safety.

**TABLE 1:** Safety of the healthcare system (*n* = 80).

Item	Strongly disagree		Disagree		Neutral		Agree		Strongly agree	
	*n*	%	*n*	%	*n*	%	*n*	%	*n*	%
Most healthcare workers make errors	1	1.3	5	6.3	24	30.0	39	48.8	11	13.8
In my country there is a safe system of healthcare for patients	13	16.3	29	36.3	22	27.5	11	13.8	5	6.3
Physiotherapy error is very common	4	5.0	28	35.0	37	46.3	9	11.3	2	2.5
It is very unusual for patients to be given the wrong therapy	3	3.8	19	23.8	27	33.8	23	28.7	8	10.0
Healthcare staff receive training in patient safety	5	6.3	10	12.5	26	32.5	28	35.0	11	13.8
About one in 10 hospital patients across the world will experience some kind of adverse event	1	1.3	11	13.8	25	31.3	27	33.8	16	20.0

**TABLE 2:** Personal influence and attitudes to patient safety (*n* = 80).

Personal influence and attitudes to patient safety	Strongly disagree		Disagree		Neutral		Agree		Strongly agree	
	*N*	%	*N*	%	*N*	%	*N*	%	*N*	%
Telling others about an error I made would be easy	6	7.5	21	26.3	33	41.3	16	20.0	4	5.0
It is easier to find someone to blame rather than focus on the causes of error	5	6.3	11	13.8	26	32.5	30	37.5	8	10.0
I am confident about speaking to someone who is showing a lack of concern for a patient’s safety	4	5.0	13	16.3	14	17.5	40	50.0	9	11.3
I know how to talk to people who have made an error	1	1.3	11	13.8	31	38.8	32	40.0	5	6.3
I am always able to ensure that patient safety is not compromised	1	1.3	3	3.8	32	40.0	36	45.0	8	10.0
I believe that filling in reporting forms will help to improve patient safety	2	2.5	3	3.8	18	22.5	39	48.8	18	22.5
I am able to talk about my own errors	3	3.8	12	15.0	20	25.0	32	40.0	13	16.3
By concentrating on the causes of incidents I can contribute to patient safety	1	1.3	5	6.3	12	15.0	42	52.5	20	25.0
If I keep learning from my mistakes, I can prevent incidents	0	0	2	2.5	7	8.8	34	42.5	37	46.3
Acknowledging and dealing with my errors will be an important part of my job	2	2.5	0	0	7	8.8	30	37.5	41	51.2
It is important for me to learn how best to acknowledge and deal with my errors by the end of my training	0	0	2	2.5	3	3.8	30	37.5	45	56.3

The majority 58 (72.5%) of respondents had a high level of knowledge with regards to safety in the workplace, 22 (27.5%) respondents had a moderate level, and 78 (97.5%) respondents had a moderate level of overall knowledge on patient safety. Tables 3 shows the outcomes on safety of the workplace and overall knowledge on patient safety. There was no significant association (*p* = 0.712) between the level of study and knowledge of clinical year physiotherapy students on patient safety (Table 4).

**TABLE 3:** Safety of the workplace (*n* = 80).

Safety of the workplace	Strongly disagree		Disagree		Neutral		Agree		Strongly agree	

	*N*	%	*N*	%	*N*	%	*N*	%	*N*	%
The physiotherapy assistants will be committed to identifying and addressing patient safety risks	4	5.0	10	12.5	24	30.0	32	40.0	10	12.5
The physiotherapy assistants will not criticise me for making mistakes	10	12.5	31	38.8	21	26.3	15	18.8	3	3.8
The physiotherapists will be committed to identifying and addressing patient safety risks	0	0	4	5.0	11	13.8	37	46.3	28	35.0
The physiotherapists will not criticise me for making mistakes	19	23.8	39	48.8	13	16.3	5	6.3	4	5.0
Managers in the healthcare system will make it easy to report errors	3	3.8	10	12.5	33	41.3	24	30.0	10	12.5
Managers in the healthcare system will be more interested in meeting performance targets than in patient safety	9	11.3	17	21.3	24	30.0	21	26.3	9	11.3
Managers in the healthcare system will expect us to focus on patient safety	0	0	3	3.8	21	26.3	38	47.5	18	22.5
Being open and honest about the mistakes I make will be acceptable at my workplace	1	1.3	6	7.5	32	40.0	27	33.8	14	17.5
Admitting an error I had made would lead to just and fair treatment by management	2	2.5	8	10.0	23	28.7	31	38.8	16	20.0

**TABLE 4:** Chi-square analysis of the association between respondents’ level of study and the level of knowledge on patient safety (*n* = 80).

Level of study	Composite level of knowledge				Pearson’s chi-square value	Degree of freedom	*p* value
	Low level	Moderate level	High level	Total			
Level 300	0	49	1	50	-	1	0.712
Level 400	0	29	1	30	0.137	-	-

**Total**	**0**	**80**	**2**	**80**	**-**	**-**	**-**

## Discussion

The moderate level of knowledge of patient safety amongst clinical year physiotherapy students is positive. However, there appears to be a lack of depth in the knowledge of patient safety acquired in the undergraduate programme. In the training of physiotherapists, knowledge can be attained from a formal curriculum or from job training. It appears that knowledge regarding patient safety was acquired from clinical rotations because it is not included in any course in the undergraduate curricula of both universities, which probably accounts for respondents’ moderate level of knowledge. On the contrary, a similar study conducted amongst nurses show that patient safety was well integrated in their overall training, and clinical aspects of patient safety were well covered in the education programme, hence the majority had a high knowledge of patient safety (Usher et al. 2017). Their findings suggest that the students were willing to study the concept of patient safety if they are specifically taught in class.

According to Hutchinson and Jackson (2013) despite concerns for patient safety, silence or inaction is more likely in unsupportive environments. Clinical year physiotherapy students in our study had a fair understanding of the different types of human error, ways of speaking up about error and how to report an error which could potentially reduce silence or inaction. This finding is however at variance with reports that the rates of non-disclosure or failure to voice concern about errors or events pose patient safety risks, which was common amongst most of the health students, as shown by Castel et al (2015) and Usher et al (2017).

Enacting discretionary voice behaviour about safety concerns may challenge the status quo and established power dynamics, and is more likely to occur in blame-free work environments that support reporting and engagement with safety improvement. (Dekker & Breakey 2016)

Our respondents seem to have a high knowledge on the factors that influences patient safety. It seems that during students’ clinical rotations, the clinical supervisors teach them how to ensure patient safety. This could be the reason why there was a high level of knowledge of the factors that influences patient safety (Usher et al. 2017).

Struessel et al. (2017) report, that irrespective of the quality of training received by healthcare professionals, errors are inevitable. This may be the reason why most of the students believed healthcare workers make errors. However, the majority of the respondents disagreed that physiotherapy error is common. This could partly be, because the students are not well exposed to error and error management in patient safety and may therefore have their own definitions or assumption of what an error is and therefore, would naturally disagree that physiotherapy error is common. It could also be that students assign an incident to error only when it results in death, falls, concussions and other major effects other than minor effects like anxiety and lack of confidence (Rosenfeld 2017).

Most students agreed that physiotherapists will be committed to identifying and addressing patient safety risks; however, they disagreed that physiotherapists will not criticise them for making mistakes. This comes on the back of respondents’ high level of knowledge on safety of the workplace and suggests that the students probably receive criticisms when they make mistakes during their clinical rotations. The respondents had divergent responses as to whether managers in the healthcare system will be more interested in meeting performance targets than in patient safety. This may be as the result of differences in encounters the respondents may have had with management of the healthcare systems in different health facilities.

Although the students were in different levels of study, they had almost the same level of knowledge of patient safety. Blasiak et al. (2014) also indicated that the year or level of study was not significantly associated with medical students’ knowledge of patient safety. It could be that, because students are exposed to the concept of patient safety at the clinics, irrespective of their level of study, they attain an appreciable level of knowledge, which probably explains why there is no significant association between the levels of knowledge amongst the participants.

## Conclusion

Clinical year physiotherapy students in Ghana have a moderate level of knowledge of patient safety. They give relevance to the subject and hope to acquire the level of knowledge they need before practicing. Further studies could be carried out to determine the level of knowledge of patient safety amongst practicing physiotherapists in Ghana.

## Appendix 1

### World Health Organization Patient Safety Curriculum Guide for Medical Schools

#### BACKGROUND INFORMATION

Sex……………………     Age………….           Year/Level of study……………….

Institution: UG/UHAS (please underline)           Nationality…………………………

#### Section 1 Error and Patient Safety

***Please circle the number that best describes your level of knowledge for each item.***

**Table:**

What is your level of knowledge regarding:	**Low**				**High**
**1.** Different types of human error?	1	2	3	4	5
**2.** Factors contributing to human error?	1	2	3	4	5
**3.** Factors influencing patient safety?	1	2	3	4	5
**4.** Ways of speaking up about error?	1	2	3	4	5
**5.** What should happen if an error is made?	1	2	3	4	5
**6.** How to report an error?	1	2	3	4	5
**7.** The role of healthcare organisations (e.g. hospitals, in-home services) in error reporting?	1	2	3	4	5

#### Section 2 Safety of the Healthcare System

***Please circle the number that best describes your level of agreement for each statement.***

1 = Strongly disagree     2 = Disagree     3 = Neutral     4 = Agree     5 = Strongly agree

**Table:**

**8.** Most healthcare workers make errors.	1	2	3	4	5
**9.** In my country there is a safe system of healthcarefor patients.	1	2	3	4	5
**10.** Physiotherapy error is very common.	1	2	3	4	5
**11.** It is very unusual for patients to be given thewrong therapy.	1	2	3	4	5
**12.** Healthcare staff receives training in patient safety.	1	2	3	4	5
**13.** About one in 10 hospital patients across the world will experience some kind of adverse event.	1	2	3	4	5

#### Section 3 Personal Influence over Safety

***Thinking about your own ability to influence patient safety, please circle the number that best describes your personal view for each statement.***

1 = Strongly disagree     2 = Disagree     3 = Neutral     4 = Agree     5 = Strongly agree

**Table:**

**14.** Telling others about an error I made would be easy.	1	2	3	4	5
**15.** It is easier to find someone to blame rather than focus on the causes of error.	1	2	3	4	5
**16.** I am confident about speaking to someone who is showing a lack of concern for a patient’s safety.	1	2	3	4	5
**17.** I know how to talk to people who have made an error.	1	2	3	4	5
**18.** I am always able to ensure that patient safety is not compromised.	1	2	3	4	5
**19.** I believe that filling in reporting forms will help to improve patient safety.	1	2	3	4	5
**20.** I am able to talk about my own errors.	1	2	3	4	5

#### Section 4 Personal Attitudes to Patient Safety

***Thinking about your personal attitudes with regard to patient safety, please circle the number that best describes your own attitude for each statement.***

1 = Strongly disagree     2 = Disagree     3 = Neutral     4 = Agree     5 = Strongly agree

**Table:**

**21.** By concentrating on the causes of incidents I can contribute to patient safety.	1	2	3	4	5
**22.** If I keep learning from my mistakes, I can prevent incidents.	1	2	3	4	5
**23.** Acknowledging and dealing with my errors will be an important part of my job.	1	2	3	4	5
**24.** It is important for me to learn how best to acknowledge and deal with my errors by the end of my training.	1	2	3	4	5

#### Section 5 Safety at the Workplace

***Thinking about your expectations about patient care when you begin working, please circle the number that best describes your expectations for each statement.***

1 = Strongly disagree     2 = Disagree     3 = Neutral     4 = Agree     5 = Strongly agree

**Table:**

**25.** The physiotherapy assistants will be committed to identifying and addressing patient safety risks.	1	2	3	4	5
**26.** The physiotherapy assistants will not criticise me for making mistakes.	1	2	3	4	5
**27.** The physiotherapists will be committed to identifying and addressing patient safety risks.	1	2	3	4	5
**28.** The physiotherapists will not criticise me for making mistakes.	1	2	3	4	5
**29.** Managers in the healthcare system will make it easy to report errors.	1	2	3	4	5
**30.** Managers in the healthcare system will be more interested in meeting performance targets than in patient safety.	1	2	3	4	5
**31.** Managers in the healthcare system will expect us to focus on patient safety.	1	2	3	4	5
**32.** Being open and honest about the mistakes I make will be acceptable at my place of work.	1	2	3	4	5
**33.** Admitting an error I had made would lead to just and fair treatment by management.	1	2	3	4	5

***Thank you for taking the time to complete this questionnaire***

## Appendix 2

### Scoring of the World Health Organization Patient Safety Curriculum Guide for Medical Schools questionnaire

#### Error and Patient Safety

In order to measure the respondents’ knowledge of patient safety, the Likert scale was used to score all the items under error and patient safety. A score of 1 was assigned to ‘very low’, 2 to ‘low’, 3 to ‘moderate’, 4 to ‘high’ and 5 to ‘very high’. These provided a generalised scale for measuring the knowledge of patient safety. The scale for measurement was classified as follows:

1. Low level of knowledge (7–14)
2. Moderate level of knowledge (15–27)
3. High level of knowledge (28–35)

#### Safety of the Healthcare system

To measure the respondents’ knowledge on safety of the healthcare system and the workplace, a scale of measurement was computed using all the questions asked under sections 2 to 5 of the questionnaire. A score of 1 was assigned to ‘strongly disagree’, 2 for ‘disagree’, 3 for ‘neutral’, 4 for ‘agree’ and 5 for ‘strongly agree’. The scale for measurement was classified as follows:

1. Low level of knowledge (6–12)
2. Moderate level of knowledge (13–18)
3. High level of knowledge (19–30)

#### Personal influences and attitudes to patient safety

To measure the respondents’ knowledge on their personal influence over safety, a scale of measurement was computed using all the questions asked under sections 2 to 5 of the questionnaire. A score of 1 was assigned to ‘strongly disagree’, 2 for ‘disagree’, 3 for ‘neutral’, 4 for ‘agree’ and 5 for ‘strongly agree’. The scale for measurement was classified as follows:

1. Low level of knowledge (11–22)
2. Moderate level of knowledge (23–33)
3. High level of knowledge (34–55)

The scale for measuring respondents’ knowledge on safety of the workplace was classified as follows:

1. Low level of knowledge (9–18)
2. Moderate level of knowledge (19–27)
3. High level of knowledge (28–45)

#### Overall knowledge level

The scale for measuring the respondents’ overall level of knowledge on patient safety was classified as follows:

1. Low level of knowledge (33–66)
2. Moderate level of knowledge (67–131)
3. High level of knowledge (132–165)
